# Rabies virus uniquely reprograms the transcriptome of human monocyte-derived macrophages

**DOI:** 10.3389/fcimb.2023.1013842

**Published:** 2023-01-31

**Authors:** Carmen W.E. Embregts, Annelieke S. Wentzel, Alexander T. den Dekker, Wilfred F.J. van IJcken, Ralph Stadhouders, Corine H. GeurtsvanKessel

**Affiliations:** ^1^ Department of Viroscience, Erasmus Medical Center, Rotterdam, Netherlands; ^2^ Orthopaedic Biomechanics, Department of Biomedical Engineering and Institute for Complex Molecular Systems (ICMS), Eindhoven University of Technology, Eindhoven, Netherlands; ^3^ Center for Biomics, Erasmus Medical Center, Rotterdam, Netherlands; ^4^ Department of Pulmonary Medicine, Erasmus Medical Center, Rotterdam, Netherlands; ^5^ Department of Cell Biology, Erasmus Medical Center, Rotterdam, Netherlands

**Keywords:** rabies virus (RABV), *Lyssavirus*, macrophage polarization, innate immunity, transcriptomics, RNA sequencing

## Abstract

Macrophages are amongst the first immune cells that encounter rabies virus (RABV) at virus entry sites. Activation of macrophages is essential for the onset of a potent immune response, but insights into the effects of RABV on macrophage activation are scarce. In this study we performed high-throughput sequencing on RNA extracted from macrophages that were exposed to RABV for 48 hours, and compared their transcriptional profiles to that of non-polarized macrophages (M0), and macrophages polarized towards the canonical M1, M2a and M2c phenotypes. Our analysis revealed that RABV-stimulated macrophages show high expression of several M1, M2a and M2c signature genes. Apart from their partial resemblance to these phenotypes, unbiased clustering analysis revealed that RABV induces a unique and distinct polarization program. Closer examination revealed that RABV induced multiple pathways related to the interferon- and antiviral response, which were not induced under other classical polarization strategies. Surprisingly, our data show that RABV induces an activated rather than a fully suppressed macrophage phenotype, triggering virus-induced activation and polarization. This includes multiple genes with known antiviral (e.g. APOBEC3A, IFIT/OAS/TRIM genes), which may play a role in anti-RABV immunity.

## 1 Introduction

Rabies is a zoonotic encephalitis that is caused by members of the genus *Lyssavirus*, -ssRNA viruses of the *Rhabdoviridae* family. Around 59 000 human cases are reported every year in over 150 countries (Hampson et al., 2015; WHO, 2018), but the true burden is likely higher due to massive underreporting and diagnostic difficulties (Hemachudha et al., 2002; Taylor et al., 2017). While 99% of rabies cases are caused by bites of rabies virus (RABV) infected dogs (WHO, 2018), other lyssaviruses, transmitted by other mammals, have caused human fatalities as well (Warrell and Warrell, 2004). Development of the disease is preventable by administrating post-exposure prophylaxis (consisting of rabies vaccine and in non-vaccinated individuals, combined with rabies immunoglobulins) within 48 hours after being exposed to the virus. However, these treatments are expensive and mostly unavailable in rabies-endemic areas (Meslin, 2005; Sudarshan et al., 2007). The disease has a case-fatality rate that approaches 100%, as there are no treatment options available after onset of neurological symptoms. This makes rabies the deadliest zoonosis worldwide.

The high case-fatality rate can largely be attributed to the fact that RABV is able to reach the central nervous system (CNS) without inducing a strong local immune response (Schnell et al., 2010; Yamaoka et al., 2013) and most often also without triggering systemic neutralizing antibodies (Kasempimolporn et al., 1991; Noah et al., 1998). RABV uses multiple mechanisms to evade and suppress the immune system, and major immune evasive mechanisms have been identified (Lafon, 2011; Ito et al., 2016; Katz et al., 2017). The absence of a strong local immune response suggests that RABV might suppress immune cells present at the initial site of infection. However, the effects of RABV on resident or recruited immune cells in skin or muscle remain largely unstudied.

Macrophages are amongst the first cells that encounter pathogens at injured sites, to which they are recruited in large numbers. They play an essential role in the onset of a specific immune response by clearing pathogens, presenting antigens and producing multiple pro-inflammatory cytokines (Mosser and Edwards, 2008; Koh and Dipietro, 2019). Furthermore, macrophages are versatile cells that can polarize toward a whole spectrum of different functional phenotypes in response to stimuli (Xue et al., 2014), which are traditionally divided into pro-inflammatory (M1), wound healing and tissue repair (M2a), or regulatory/anti-inflammatory (M2c) profiles (Mosser and Edwards, 2008). Many viruses are known to steer macrophage polarization into a direction that is beneficial for the virus (Sang et al., 2015). A strong influx of macrophages has been reported in RABV-inoculated sites as well (Charlton and Casey, 1981), but it remains largely unknown if RABV exerts mechanisms to affect macrophage polarization, mainly because of the limited translatability of available studies due to the use of lab-adapted (non-wild-type) RABV strains, cell lines or cells of non-human origin (Turner and Ballard, 1976; Ray et al., 1995; Nakamichi et al., 2004; Kip et al., 2017). We recently showed that RABV is able to bind to the surface of human primary monocyte-derived macrophages, leading to induction of the cholinergic anti-inflammatory pathway and a decreased T-cell activation (Embregts et al., 2021). In addition, exposure of macrophages to RABV for a period of 48 hours induced elevated expression of the activation markers PD-L1 and CD163, hinting that RABV can also affect macrophage activation (Embregts et al., 2021).

In the presented study we characterized the effects of RABV on macrophage polarization in detail by performing transcriptome analysis of human monocyte-derived macrophages stimulated with street RABV, and compared the induced transcriptional profile to the canonical macrophage phenotypes induced by LPS and IFN-γ (M1), IL-4 (M2a), and IL-10 (M2c). We found that RABV induced a transcriptional profile that was distinct from canonical M0, M1, M2a or M2c profiles. Based on the expression levels of signature M1/M2a/M2c genes, RABV macrophages showed the highest resemblance to M1 macrophages. RABV macrophages also displayed a set of strongly expressed genes that were not induced in the other phenotypes, and pathway enrichment analysis showed that these genes were involved in viral responses, interferon production and cytokine signaling. Altogether, our data shows that prolonged exposure of RABV does not completely suppress macrophages, but instead induces intrinsic phenotypical changes in human monocyte-derived macrophages, resulting in a unique transcriptomic polarization program.

## 2 Materials and methods

### 2.1 Monocyte isolation and macrophage maturation

Monocytes were isolated from buffy coats from healthy, non-rabies vaccinated blood donors, and were differentiated towards macrophages as described before (Embregts et al., 2021). Buffy coats were obtained from the Sanquin blood bank (Rotterdam, the Netherlands), in accordance with the Dutch law on acquirement of blood and blood components (BWBR0017977), the European directives 2002/98/EC, 2004/33/EC and 2005/61/EC, the General Data Protection Regulation (GDPR), and after obtaining written informed consent for research use of donated blood. The research use of donated blood was approved by the Sanquin Ethical Advisory Board. Briefly, blood mononuclear cells (PBMCs) were isolated from the donors blood by density centrifugation using Ficoll Paque PLUS (GE Healthcare), after which the monocytes were magnetically sorted using CD14^+^ beads (Miltenyi Biotec). Purity of the sorted populations was confirmed using a BD Lyric flow cytometer (BD Biosciences). Only monocyte samples with a purity of >95% were used for macrophage maturation.

Monocytes (100.000 cells per well of a 96-well plate) were maturated for 6 days in maturation medium (RPMI-1640 medium (Lonza) supplemented with 10% pooled human serum (Sanquin), 1% (v/v) GlutaMAX (Gibco), and 20 ng/mL macrophage colony-stimulating factor (M-CSF, R&D Systems)), which was replaced every other day. Cells were maintained at 37°C with 5% CO_2_.

### 2.2 Polarization of macrophages

Mature macrophages were polarized into M1, M2a, M2c macrophages or the “RABV-exposed” phenotype (from now on referred to as the RABV phenotype or RABV macrophages) by incubation for 48 hours with IFN-γ (20 ng/mL, R&D Systems) and LPS (100 ng/mL, Sigma Aldrich), IL-4 (20 ng/mL, R&D Systems), IL-10 (20 ng/mL R&D Systems), or with RABV (MOI of 10), respectively. Human monocyte-derived macrophages are not susceptible to RABV, and exposure of human macrophages to RABV does not lead to infection, as we reported in a previous study (Embregts et al., 2021). The RABV strain used in this study was a low-passage (second passage, P2) silver-haired bat rabies virus (SHBRV), a street RABV strain causing human infections in North America. The virus was propagated in SK-N-SH human neuroblastoma cells (Embregts et al., 2021) and virus concentration was determined by the median tissue culture infective dose (TCID_50_) endpoint dilution method of Reed and Muench (Reed and Muench, 1938). The virus stocks were sequenced before use, to confirm that no culture-related genetic adaptations had occurred. In addition, cytokine levels in the SHBRV stocks were quantified using the Legendplex 13-plex human antiviral kit, to verify that polarization of macrophages was not induced by cytokines present in the virus stock. All samples were measured in triplicate and according to the manufacturer guidelines. This analysis showed that the concentration of the cytokines in the culture medium during polarization, corrected for the dilution of the virus stock, was below 10 pg/mL. Moreover, IFN- γ and IL-10, inducers of M1 and M2c macrophages, respectively, were detected at negligible amounts (**Supplementary Table 1**.)

### 2.3 RNA isolation

At the end of the 48 hour polarization period, RNA was isolated from the macrophages using the RNeasy micro kit (Qiagen), according to the manufacturer’s guidelines and including the on-column DNAse digestion treatment. In short, plates containing the macrophages were washed twice with warm PBS, after which the macrophages were lysed by adding 75 μL lysis buffer to each well. Complete lysis was ensured by vortexing the collected lysate, after which the standard protocol was continued. Eluted RNA was stored at -80°C until further processing. Successful macrophage polarization was verified by flow cytometry from replicate wells as described before (Embregts et al., 2021).

### 2.4 RNA sequencing and mapping

Integrity, purity and concentration of the extracted RNA was determined using a BioAnalyzer and total RNA Pico chips (Agilent). cDNA libraries were prepared from the isolated RNA using the Smart-seq2 method (Picelli et al., 2013). Libraries were prepared for all five conditions (M0, M1, M2a, M2c, RABV) from three individual donors. Obtained libraries were sequenced on an Illumina HiSeq2500 sequencer, generating single-end reads of 50 base pairs. Subsequently, Illumina adapter sequences and poly-A stretches were trimmed and remaining sequences were aligned to the reference human genome assembly GRCh38 using HISAT2 v2.1.0 (Kim et al., 2019). Counts of the alignments were determined using htseq-count v.0.11.2 (Anders et al., 2015). Gene details were extracted from the NCBI database using the R packages rentrez (Winter, 2017) and biomaRt (Durinck et al., 2009). Pearson correlation coefficients (PCC) were calculated on the log-transformed normalized counts to use as an initial investigation of the inter-sample relationships.

### 2.5 Differential gene expression analysis

Differentially gene expression analysis was performed with DESeq2 v1.28.1 (Love et al., 2014) and the R statistical software version 4.0.2 (R.Core.Team, 2020). Most data manipulations were performed with functions of the R packages dplyr (Wickham et al., 2021) and matrixStats (Bengtsson, 2021). Minimal filtering was applied to exclude genes that had zero counts in all datasets. Paired analysis was performed to correct for donor-to-donor variation, and non-polarized M0 macrophages were included as controls. The differential gene expression analysis included Benjamini & Hochberg corrections and gene expression levels were considered significantly different when *p*_adjusted_ < 0.05. Genes were considered differentially expressed when log_2_ fold change < -1 or > 1. An additional threshold of > 0.5 reads per kilo base per million mapped reads (RPKM) was used to only include substantially expressed genes in downstream analyses. The expression levels of a selected set of genes were validated by quantitative PCR (qPCR), using SYBR Green PCR Mastermix (Applied Biosystems) and a 7500 Real Time PCR Cycler (Applied Biosystems). Expression levels were normalized against the housekeeping gene TBP. ΔCt values of the selected genes and the used primers are shown in **Supplementary Figure 1** and **Supplementary Table 2**, respectively.

### 2.6 Pathway enrichment analysis

Pathway enrichment analysis was performed with the web-based gene annotation and analysis tool Metascape (Zhou et al., 2019), using a minimum overlap of 3, a *p*-value cutoff of 0.01 and a minimum enrichment of 1.5.

### 2.7 Data visualization

All visualization plots (heatmaps, volcano plot) were generated in R using ggplot2 (Wickham, 2016) and ggalt, unless stated otherwise. Principal component analysis plots were made using PCAtools (Blighe and Lun, 2021), on the logCPM (counts per million reads) values for the top 1000 genes with the highest standard deviation throughout the dataset. The lowest 10% of hits were removed based on variation. Venn diagrams were calculated and visualized using Venn (Dusa, 2020). Clustered heatmaps were generated with pheatmap (Kolde, 2021) with color schemes generated in RColorBrewer (Neuwirth, 2014).

## 3 Results

### 3.1 RABV macrophages highly express both M1 and M2a/M2c signature genes

**Figure 1:**
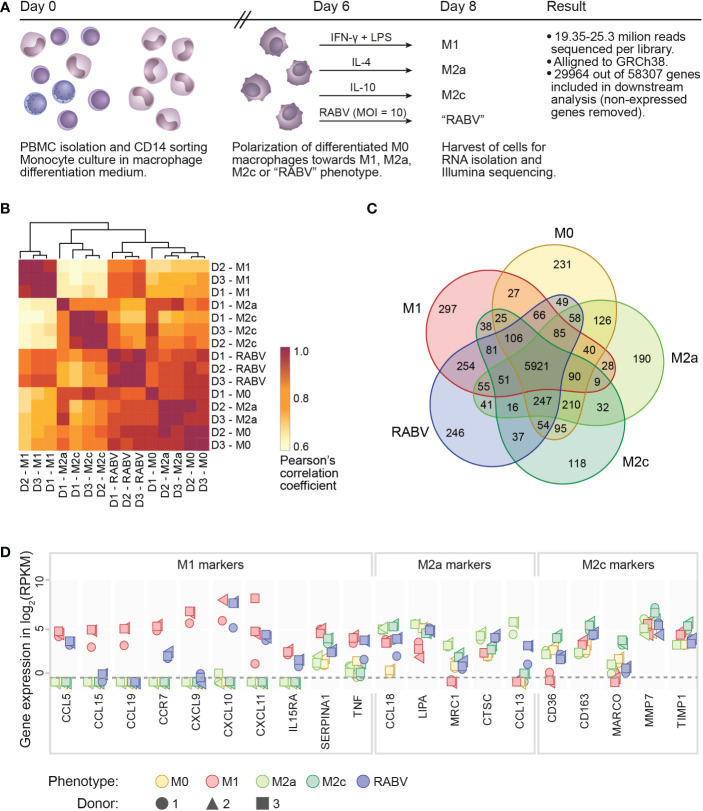
Experimental layout, initial analysis on sample variation and confirmation of the polarization model. A schematic overview of the presented transcriptome study is shown in **(A)**, indicating the experimental timeline, the experimental groups and the number of reads and genes retrieved from the Illumina sequencing. Clustering of the individual samples was based on calculated Pearson’s correlation coefficients **(B)**. The number of substantially expressed genes (mean RPKM > 0.5 of the three individual samples per phenotype), is presented in the Venn diagram in **(C)**. The expression of signature M1, M2a or M2c genes in all individual samples is shown, in log_2_ RPKM, in **(D)**. The dotted line indicates the threshold for substantial expression, at 0.5 RPKM or -0.3 log_2_. For clarity, samples with an expression level of <0.5 RPKM are all placed below this line.

High-throughput sequencing was performed on RNA extracted from human monocyte-derived macrophages (from n=3 individual donors) that were polarized into M0, M1, M2a, M2c or “RABV” phenotypes (**Figure 1A**), in order to get novel insights into the effects of RABV on macrophage activation and polarization. 19.35 to 25.3 million reads were sequenced per sample and after excluding genes with zero counts, 29964 genes were included for downstream analysis. We first evaluated the variation and correlation across the individual samples by plotting a Pearson’s correlation coefficients (r) matrix based on all 29964 genes (**Figure 1B**). The matrix shows that the M1 samples form a cluster that is distinct from a second larger cluster that contains all M0, M2a, M2c and RABV samples, indicating that M1 macrophages have the most unique gene expression profile when comparing all five phenotypes included in this study.

When focusing on the individual macrophage phenotypes we can see that all the three donors from the M1- and RABV-stimulated samples form two clear clusters, while M2a, M2c and M0 phenotypes showed less distinct transcriptional responses. Variation between samples is mostly caused by the polarization treatment and not by the individual donors, with excellent reproducibility across biological replicates (Pearson’s r > 0.90).

The number of substantially expressed genes (mean RPKM of > 0.5 per phenotypic group, total of n= 8986 genes), and the overlap with the other phenotypes, is shown in a Venn diagram (**Figure 1C**). The diagram shows that a large proportion of substantially expressed genes (5921/8986, 65.9%) are expressed in all macrophage phenotypes. Next to these common genes, each phenotype expresses a set of genes that are only expressed in the indicated phenotype, and not or at very low levels (RPKM < 0.5) in the other four phenotypes. These genes were therefore considered to be phenotype-specific in the context of this study. M1 macrophages have most phenotype-specific genes (297), followed by RABV (246), M0 (231), M2a (190) and M2c (118). In terms of overlapping signatures, we see that 254 expressed genes are shared only between RABV and M1 conditions (uniquely shared). This number is higher than the number of uniquely shared genes of RABV and M0 (49), M2a (41) and M2c (37). While the Pearson’s r matrix that was calculated on all genes showed that RABV most resembles the M0, M2a and M2c phenotypes, this overlap in substantially expressed genes indicates that RABV macrophages might show a closer resemblance to M1 macrophages than to the other phenotypes included in our study. Apart from the genes that were expressed in multiple phenotypes, 246 genes were found to be only substantially expressed in RABV macrophages (summarized in **Supplementary Table 3**). Among these genes are the Toll-Like receptors TLR3 and TLR7, suggesting that RABV is recognized by human macrophages through these receptors, leading to upregulation of the TLR3 and TLR7 genes.

The polarization of M0 macrophages into M1, M2a and M2c macrophages was verified next, by selectively examining expression levels of M1 or M2 signature genes (**Figure 1D**). As all signature genes were most strongly expressed in the corresponding phenotype, we concluded that polarization of M1, M2a and M2c macrophages was successful. In agreement with the clustering of the individual samples in **Figure 1B**, we observed some donor-to-donor variation. RABV macrophages express relatively high levels of multiple M1 signature genes (CCL5, CCR7, CXCL10, CXCL11, IL15RA, TNF), but also of some M2a signature genes (CCL18, CTSC and CCL13) and the M2c signature gene CD163 (**Figure 1D**). This indicates that RABV does not polarize human macrophages towards one single classical phenotype.

### 3.2 RABV induces a macrophage phenotype that is distinct from canonical M1/M2 phenotypes

**Figure 2:**
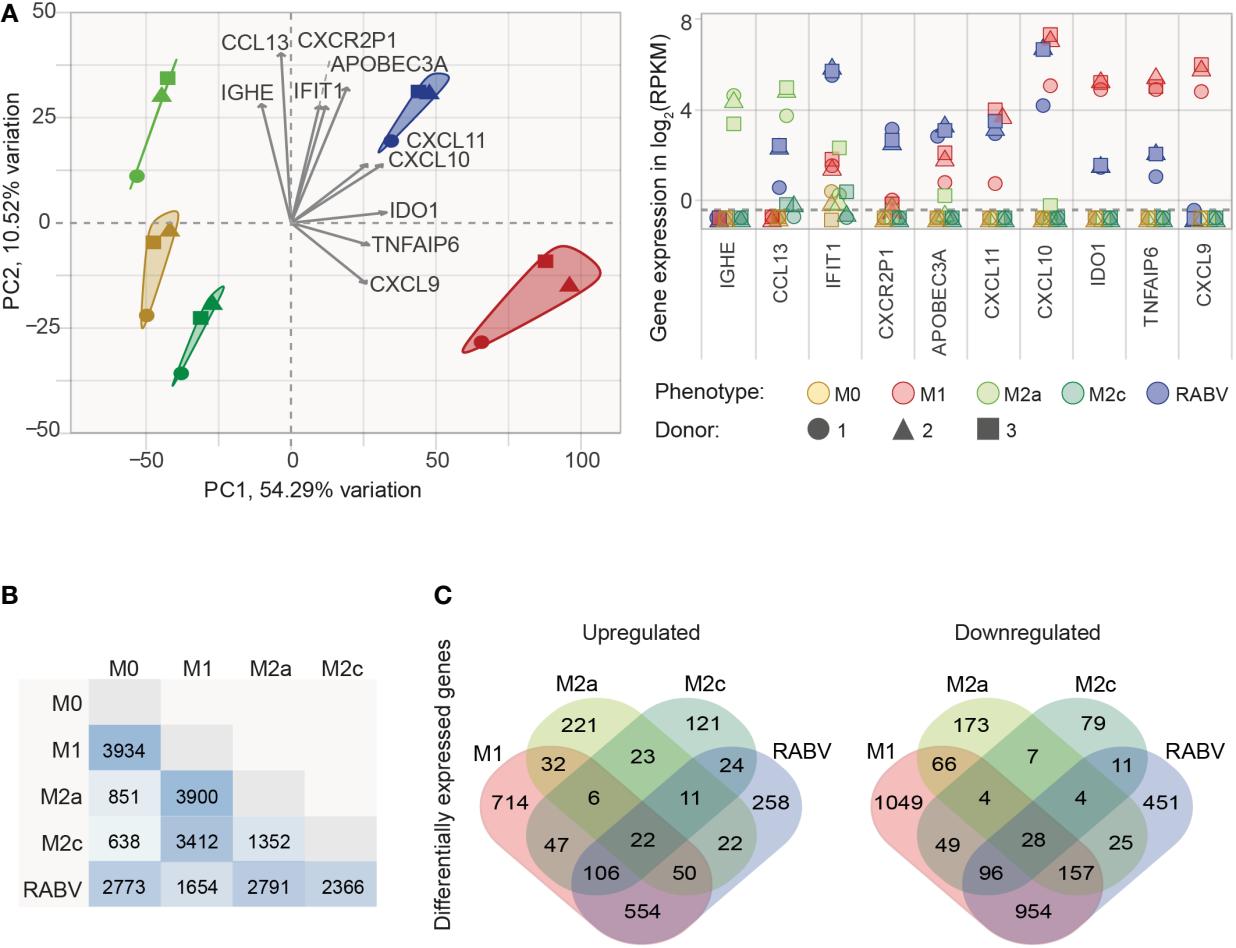
Initial analysis of the similarities and differences between the M0, M1, M2a, M2c and RABV macrophage gene expression profiles. All genes that are substantially expressed in at least one of the phenotypes (mean RPKM > 0.5) were submitted in a principal component analysis **(A)**, showing the two components responsible for the majority of variation (PC1 and PC2) and the top 10 active variables that contribute the most to this variation (highest cos_2_ values, grey arrows). The expression levels of these 10 genes for every individual samples in shown in the right graph, in log_2_ RPKM. The number of differentially expressed genes (DEGs, *p_adj_* < 0.05, log_2_ fold change < -1 or > 1) between the five different macrophage phenotypes is shown in **(B)**, as well as the number of upregulated or downregulated DEGs when compared to M0 **(C)**.

After confirming that our *in vitro* set-up resulted in successful macrophage polarization, we set out to investigate the (dis)similarity of the different macrophage phenotypes. To this end, all genes that were substantially expressed in at least one phenotype (mean RPKM > 0.5, resulting in n= 8986 genes) were used in a principal component analysis (PCA, **Figure 2A**), showing the two components responsible for the majority of variation in the dataset (PC1 and PC2 on the x- and y-axis, respectively) and the 10 variables that contributed the most to this variation (grey arrows, with expression levels of all individual samples plotted in the right panel of **Figure 1A**). The figure shows that all five macrophage phenotypes form distinct clusters. Genes that contributed the strongest to the RABV cluster included: 1) APOBEC3A, a cytidine deaminase with a suspected role in restricting RNA virus infection, 2) the interferon-induced protein IFIT1, the CXC chemokine receptor pseudogene 1 that is involved in dampening the pro-inflammatory response, and 3) the inflammatory chemokines CXCL10 and CXCL11. The expression levels of these four genes, together with TLR3, TLR7, the M2a signature gene CCL13 and the M2c signature gene CD163 were examined by qPCR, confirming the expression levels found by RNA sequencing (**Supplementary Figure 1**.)

Next, we investigated the differences in transcriptional profiles by differential gene expression analysis. When comparing the polarized macrophage phenotypes to the non-polarized M0 macrophages we see that the most differentially expressed genes (DEGs, *p_adj_* < 0.05, log_2_ fold change of < -1 or > 1) appear in the M1 (n= 3934) and RABV (n= 2773) phenotypes (**Figure 2B**). Less DEGs were found in the M2a (n= 851) and the M2c (n= 638) phenotypes. As expected, comparing M2a to M2c macrophages results in less DEGs (1352) than when compared to M1 (3900 and 3422 for M2a and M2c, respectively), or RABV macrophages (2791 and 2366 for M2a and M2c, respectively). Comparing the RABV phenotype to the others shows that RABV has the least DEGs when compared to M1 (n= 1654), followed by M2c (n= 2366), M0 (2773) and M2a (2791). Comparing the overlap of upregulated or downregulated DEGs between the different macrophage phenotypes compared to M0 (**Figure 2C**) shows a pattern where 1) all five phenotypes have a set of genes that is up- or downregulated solely in the specified phenotype and 2) regulation of genes in RABV-exposed macrophages shows the closest resemblance to M1 macrophages, given the highest number of overlapping down- or upregulated genes. A complete overview of all DEGs detected can be found in **Supplementary Table 4**.

### 3.3 RABV induces multiple antiviral- and interferon-related pathways in human macrophages

**Figure 3:**
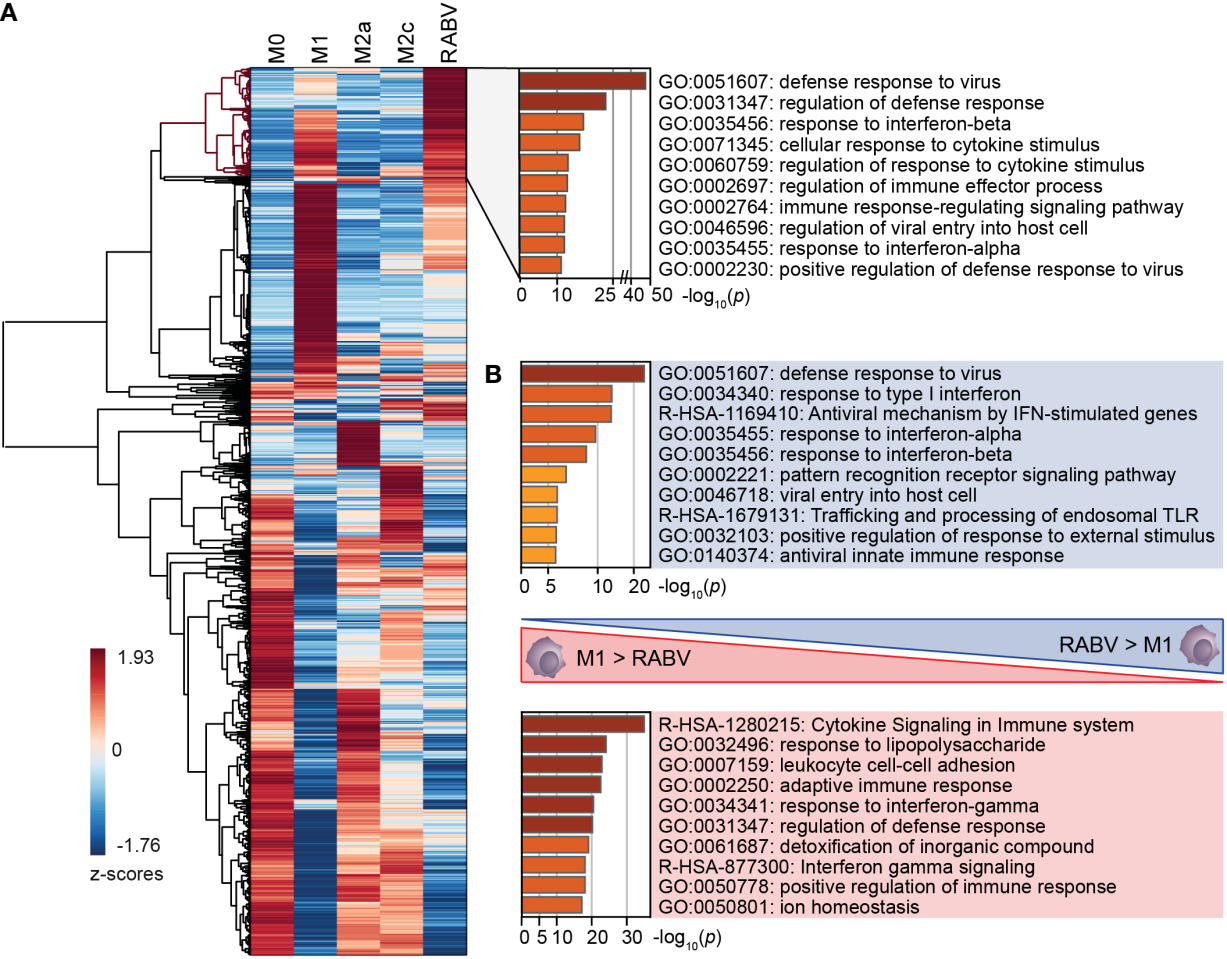
Clustering of all DEGs of the five studied macrophage phenotypes, and the major differences in enriched pathways between M1 and RABV macrophages. All genes that were differentially upregulated in at least one phenotype were clustered **(A)**. A cluster of highly upregulated genes of RABV macrophages (highlighted in red) was submitted to pathway enrichment analysis. Pathway enrichment analyses were also performed on genes that were differently upregulated in RABV macrophages but not in M1 macrophages, and vice versa **(B)**.

RABV macrophages share a large number of upregulated genes with M1, M2a and M2c macrophages, which was further investigated by performing cluster analysis on all genes that were differentially upregulated and substantially expressed in at least one of the macrophage phenotypes (*p_adj_ <*0.05, log_2_ fold change relative to M0 >1, RPKM >0.5). The resulting clustered heatmap of z-scores, indicating the relative expression of the 5105 selected genes, is shown in **Figure 3A**.

This heatmap shows a cluster of 623 genes that are most strongly expressed in RABV macrophages when compared to the other phenotypes included on our study (**Figure 3A**, dendrogram highlighted in red). Pathway enrichment analysis of this cluster shows that a large proportion of genes are involved in interferon signaling and response, and antiviral mechanisms (**Figure 3A**, right). Genes in this cluster included APOBEC3A, CASP1, multiple chemokines and receptors (CCL2, CCL4L2, CCL7, CXCR4, and CXCR2P1, the pseudogene 1 of the immune-dampening cytokine CXCR2), many interferon-induced genes (IFI6, IFI27, IFI35, IFI44, IFI44L, IFIT1, IFIT2, IFIT3, IFIT5, IFITM1, IFITM2, IFITM3, IRF2, IRF7, ISG15, ISG20, MX1, MX2), genes involved in enzymatic (viral) RNA degradation (OAS1, OAS2, OAS3), SIGLEC1, SIGLEC11 and SIGLEC14 and ten TRIM proteins. These genes indicate that exposure of human macrophages to RABV triggers an activated phenotype that involves antiviral mechanisms and a strong interferon response.

A large proportion of genes in this cluster also display a high z-value in M1-macrophages, as indicated by the red color in the heatmap. To better understand the differences between these two phenotypes we had a closer look at the genes that were upregulated (*p <*0.05, log_2_ fold change >1, RPKM >0.5) in M1 macrophages when compared to RABV macrophages, and vice versa (Complete overview of genes in **Supplementary Table 5**). Genes that are upregulated in RABV macrophages compared to M1 macrophages include APOBEC3A, TLR3, the chemokines and receptors CCL13 and CXCR4, the CD-markers CD69 and CD169, ICAM2, the immune-suppressive IL10 cytokine, interferon-stimulated genes, genes involved in enzymatic (viral) RNA degradation, SIGLEC11 and SIGLEC12, and the inhibitor of TRAF6-activation TIFAB. On the other hand, M1 macrophages displayed higher levels of multiple apolipoproteins, complement-related genes, many chemokines, interleukins, and receptors, multiple CD-markers and Fc-fragment of IgG receptor genes, guanylate binding protein genes, MHC-related genes, interferon-regulating genes, metallothionein-related genes, and solute carrier family members. The functional implications of the differences between M1 and RABV macrophages were studied by pathway enrichment analysis (**Figure 3B**). This analysis showed that genes that are upregulated in RABV macrophages when compared to M1 macrophages are mostly involved in the antiviral and interferon response, and genes that are higher expressed in M1 macrophages are more involved in cytokine signaling and response to LPS. This together indicates that the M1 macrophages display a stronger pro-inflammatory profile, characterized by the strong expression of many chemokines and interleukins, while RABV macrophages display a interferon-induced and antiviral skewed phenotype. Besides this strong interferon-induced profile, the presence of IL10 in this cluster shows that RABV also triggers anti-inflammatory or immune-suppressive pathways in human monocyte-derived macrophages.

### 3.4 RABV-macrophages, a virus-specialized macrophage phenotype?

**Figure 4:**
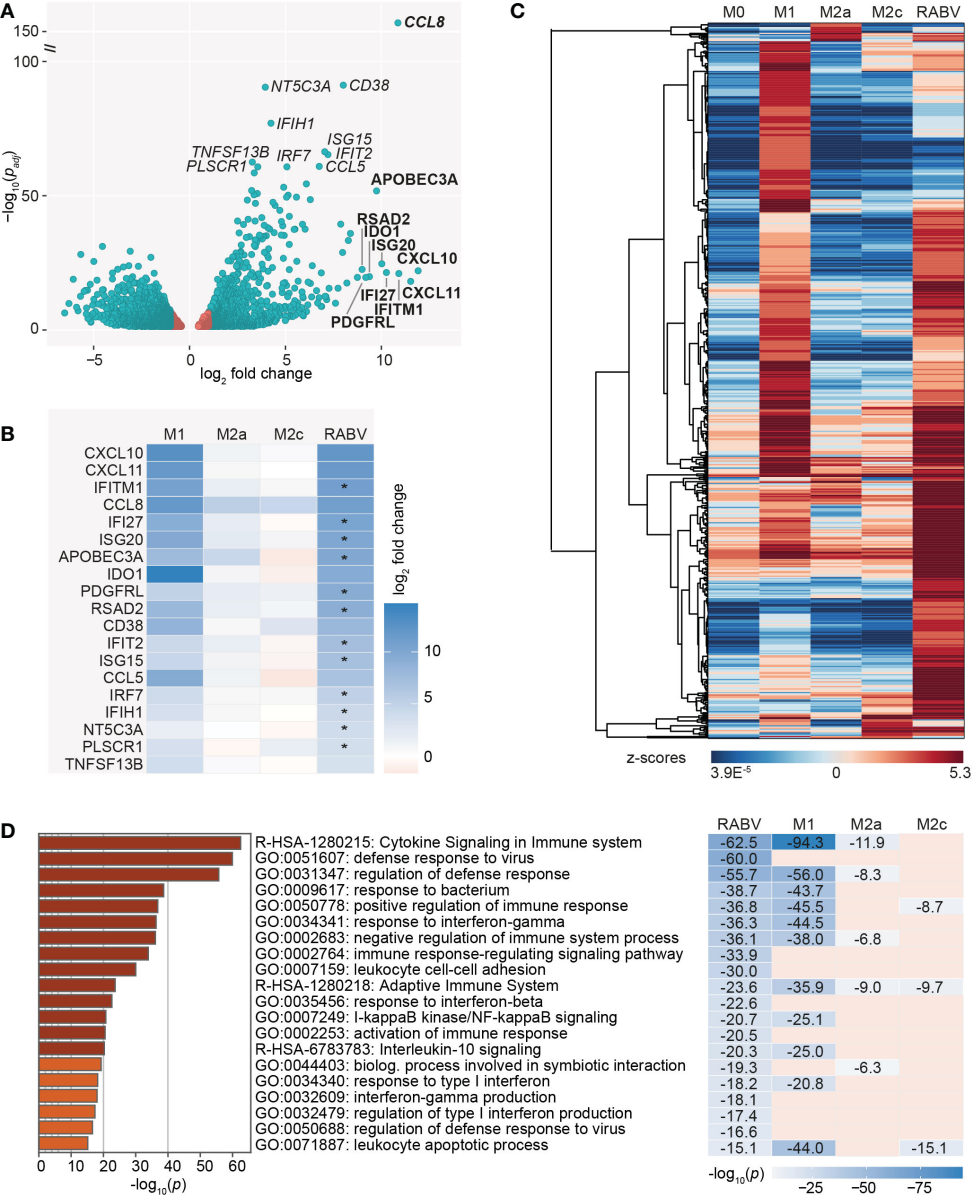
Visualization of the most significantly regulated genes of RABV-macrophages, and the induction of multiple virus response-related pathways. A volcano plot highlights the genes with the lowest adjusted *p*-value (*p_adj_*, genes in italics) and the highest upregulation (log_2_ fold change, genes in bold) of RABV macrophages compared to M0 macrophages **(A)**. Genes in red have a log_2_ fold change between -1 and 1 and are not differentially expressed. Log_2_ fold change in M1, M2a and M2c macrophages of the highlighted genes in **(A)** is shown in **(B)**. Asterisks (*) in B indicate that expression levels in RABV macrophages are significantly higher than in M1 macrophages. Clustering analysis **(C)** and pathway enrichment analysis **(D)** of the differentially and substantially expressed (RPKM > 0.5) genes of RABV macrophages. The top 20 induced pathways of RABV are compared to that of the other phenotypes, and *p*-values are shown if the specified pathway was present in the top 20 of the specified macrophage phenotype.

As a final step in the characterization of the RABV macrophage transcriptional profile we investigated the genes with significantly different (*p_adj_* < 0.05) expression levels in RABV macrophages compared to M0 (**Figure 4A**). The genes with the most significant and strongest altered expression (based on *p_adj_* or log_2_ fold change, respectively) were highlighted in italics and bold in the volcano plot, respectively, showing that the genes with the most significant changes in expression level were all upregulated genes. In agreement with the most contributing genes of the PCA, the suspected viral restriction factor APOBEC3A and the inflammatory chemokines CXCL10 and CXCL11 are amongst the top 10 most upregulated genes. The top genes also include the chemokine CCL8 and multiple interferon-induced genes and interferon regulatory factors, indicating that RABV triggers an interferon/antiviral response in human macrophages. When we compare the regulation of these highlighted genes in RABV macrophages to that of the other phenotypes (**Figure 4B**), we observe that the majority of these genes are strongly upregulated in M1 macrophages, but not in the other phenotypes.

To get a better understanding on the functional overlap between the RABV phenotype and the M1 and M2a/2c phenotypes, we calculated the relative expression (expressed in z-scores) of the upregulated and substantially expressed (RPKM >0.5) DEGs of RABV and visualized this in a heatmap (**Figure 4C**, complete overview of all 602 genes in **Supplementary Table 6**). These genes included many genes related to chemokines and cytokines, complement, caspase, antigen sensing and presentation, interferon/antiviral response and signaling. This indicates that macrophage activation, rather than complete suppression, is induced by exposing human macrophages to RABV for 48 hours. No clear gene clusters were observed, but a large proportion of genes showed high z-scores in M1 macrophages, indicating that they are higher expressed in this phenotype than in the other phenotypes included in this study.

We next investigated which biological pathways were significantly enriched in RABV macrophages, using the same genes as displayed in the clustered heatmap in **Figure 4C**, in order to gain a deeper understanding on the function of the upregulated genes. The top 20 most induced pathways in RABV macrophages, based on *p*-value, as well as their presence (including *p*-value) or absence in the top 20 induced pathways of the other phenotypes, is shown in **Figure 4D**. Pathways that are strongly induced in RABV macrophages included multiple pathways involved in cytokine signaling, (regulation of the) defense response to viruses, activation or regulation of the immune response and response to interferons. Many of the pathways induced in RABV macrophages are also present in the 20 most induced pathways of other phenotypes, and the majority is shared with M1 macrophages. Of all pathways that are shared between RABV and M1, M1 macrophages have a lower *p*-value, indicating that the pathways are more strongly induced in M1 macrophages than in RABV macrophages. Pathways that are highly induced in RABV, but not in the other phenotypes, include multiple pathways in the defense against viruses and related interferon response, and activation and regulation of the immune response.

Altogether, the data shows that RABV induced macrophages display a unique virus-induced activation and polarization profile in human monocyte-derived macrophages. While some anti-inflammatory genes were observed to be induced in RABV macrophages, the majority of induced genes are involved in the pro-inflammatory and antiviral response. Furthermore, highlighting phenotype-specific signature genes showed that RABV macrophage share a higher number of characteristics with M1 macrophages than with M2a and M2c macrophages.

## 4 Discussion

Macrophages are among the first immune cells that encounter pathogens and play an important role in activating the immune system. However, many viruses developed mechanisms to subvert the M1 pro-inflammatory cytokine response by enhancing polarization towards the M2 phenotype. In the presented study we used high-throughput mRNA sequencing to study transcriptional changes in human macrophages that were exposed to RABV for 48 hours, a time point that is commonly used to study macrophage polarization.

Examination of signature M1, M2a and M2c genes showed that RABV macrophages display a mixed transcriptional profile that included a strong interferon-induced and antiviral profile but also included signature M1, M2a and M2c genes. Upregulation of the M1-associated pro-inflammatory chemokines CXCL10 (IP-10) and CXCL11 (IP-9) was observed before in brains of RABV-infected mice (Zhang et al., 2016), RABV-infected murine macrophage cell lines (Nakamichi et al., 2004) and RABV-exposed human macrophages (Embregts et al., 2021). While multiple M1-associated genes were found to be also highly induced in RABV macrophages (CXCL10, CXCL11, IL15RA, TNFA) in our study, their expression levels were lower than in the LPS-induced M1 macrophages. This indicates that while the virus seems to activate human macrophages rather than strongly suppressing them, their activation is different from M1 macrophages. In agreement with our previous study, the M2c activation marker CD163 and the anti-inflammatory cytokine IL10 were expressed at higher levels in RABV macrophages than in M1, M2a and M2c macrophages (Embregts et al., 2021).

APOBEC3A and IFIT1 were highest expressed in RABV macrophages and were found to be amongst the most important genes for PCA clustering. APOBEC proteins interfere with viral replication by inhibiting reverse transcriptase activity and introducing genomic mutations by cytidine deamination (Takaori-Kondo, 2006). While the antiretroviral roles of APOBEC-3F and APOBEC-3G have been studied by multiple groups (Conticello et al., 2003; Liu et al., 2005), the role of APOBEC proteins in inhibiting RABV replication has not yet been studied. IFIT proteins are also viral restriction factors, and while IFIT1 has so far only been associated with positive-stranded RNA viruses including West Nile virus (Daffis et al., 2010) and Japanese encephalitis virus (Kimura et al., 2013), restriction of RABV replication has been reported for IFIT2 and IFIT3 (Davis et al., 2017; Chai et al., 2021). Interestingly, IFIT2 and IFIT3 showed the highest expression levels in RABV macrophages in our study when compared to the other studied phenotypes, and were also found to be highly upregulated in brains of mice with RABV infection (Zhao et al., 2011).

An increasing number of tripartite motif (TRIM) proteins is being recognized as interferon-stimulated genes (ISGs) (Nisole et al., 2005; Yap and Stoye, 2012). TRIM14 was uniquely induced in RABV macrophages, and TRIM5, TRIM21 and TRIM22 were induced in multiple macrophage groups. These TRIMs were previously found to block hepatitis C virus replication (Wang et al., 2016), and stimulate the type-I interferon defense against human immunodeficiency virus 1 (Luban, 2007), Japanese encephalitis virus (Manocha et al., 2014), adenovirus type 5 (McEwan et al., 2012) and influenza A virus (Di Pietro et al., 2013). Upregulation of multiple TRIM and IFIT genes and APOBEC3A and suggests that they might also serve a role in the resistance of human macrophages to RABV infection. Besides this, the large diversity of interferon-induced or antiviral genes induced in RABV macrophages indicates that the cells may use multiple ways of restricting virus infection and replication.

While we previously did not observe replication in human monocyte-derived macrophages, upregulation of the endosomal ssRNA-sensing receptor TLR7 does suggest that the virus is internalized and processed. TLR7 is a known innate recognition receptor for RABV and while TLR7 was found to play an essential role in germinal center formation (Liu et al., 2016; Luo et al., 2019) and controlling RABV infection (Faul et al., 2010), detection of RABV by TLR7 is also associated with enhanced neuroinflammation and immunopathology (Luo et al., 2020). Due to this delicate balance of activation, the effects of this upregulation requires functional studies. Next to TLR7, TLR3 was only upregulated in RABV macrophages, which has been previously shown to be a crucial receptor for innate recognition of RABV as well (Li et al., 2011).

Besides the strong expression of a multitude of M1 signature genes and many genes involved in the interferon- and antiviral response, few immune-suppressive or anti-inflammatory genes were found to be induced as well. CXCR2P1, the CXC-chemokine 2 receptor pseudogene 1, was 20-fold higher expressed in RABV macrophages than in the other phenotypes and was amongst the most contributing genes for the PCA. CXCR2 is involved in dampening the immune reaction in a very diverse manner, and activation of the receptor by binding of its ligand was reported to halt excessive skin inflammation (Dyer et al., 2017) and inflammable bowel disease (IBD) (Kishida et al., 2015), and promote macrophage polarization to the suppressive tumor-associated macrophage (TAM) phenotype (Di Mitri et al., 2019). TIFAB, the inhibitor of TRAF6 activation (Niederkorn et al., 2020), plays a role in inhibiting NF-κB and was found to be uniquely upregulated in RABV macrophages. Interestingly, the positive regulator of the TRAF6/NF-κB pathway, TIFA, was induced in M1 macrophages but not in RABV macrophages, suggesting that RABV favors TIFAB expression over TIFA, hereby dampening the inflammatory cytokine response. This is in agreement to our previous findings that show an inhibition of the pro-inflammatory TNF-α response production through blocking NF-κB nuclear translocation (Embregts et al., 2021). Upregulation of sialic acid-binding immunoglobulin-like lectin 11 (SIGLEC11) was found in RABV macrophages but not in the other macrophage phenotypes. This might indicate another suppressive effect of RABV, given that engagement of specific ligands with this SIGLEC has anti-inflammatory effects on human macrophage cell lines (Shahraz et al., 2015). However, besides TIFAB, SIGLEC11, and IL10, no true anti-inflammatory or suppressive profile was found in RABV macrophages.

In conclusion, we present the first transcriptomic analysis of a specific immune cell type after exposure to RABV, given that transcriptomic studies so far only focused on brain samples at the final stages of disease (Zhang et al., 2016; Koraka et al., 2018). When looking at signature genes and induced immune pathways, RABV macrophages showed more similarity to M1 macrophages than to the other studied macrophage phenotypes, but otherwise display a unique transcriptional profile that suggests that their activation program is different. Although expression levels cannot be directly linked to activity *in vitro* or *in vivo*, the upregulation of the interferon-induced genes does show that a certain degree of antiviral response is triggered upon RABV-exposure of human macrophages. Moreover, the upregulation of genes with a potential antiviral effect against RABV (APOBEC3A, IFIT-, OAS- and TRIM genes) deserves additional investigation.

Gaining thorough insights into the interactions between RABV and macrophages is essential for the understanding of RABV-induced immunosuppression and ultimately, for the development of new post-exposure prophylactic therapeutics that aid the onset of a rapid and strong immune response against RABV. Given this, it is of importance to translate the observed differences in gene expression profiles to *in vitro* and *in vivo* macrophage functioning. Follow-up experiments will include time-course *in vitro* functional assays and targeted *in vivo* studies that focus on gaining deeper insights into the interactions between macrophages and RABV, as well as on the effects of different RABV strains and lyssaviruses on human macrophages.

## Data availability statement

The datasets presented in this study can be found in online repositories. The names of the repository/repositories and accession number(s) can be found below: https://www.ncbi.nlm.nih.gov/geo/, GSE205990.

## Ethics statement

Ethical review and approval was not required for the study on human participants in accordance with the local legislation and institutional requirements. The patients/participants provided their written informed consent to participate in this study.

## Author contributions

CE and CG conceived the experiment, CE, AD and WI performed the experiment. Data analysis was performed by CE, with conceptual input from RS, AW and WI. All authors reviewed the manuscript. All authors contributed to the article and approved the submitted version
