# Supplementary material for: Rabies virus uniquely reprograms the transcriptome of human monocyte-derived macrophages
Source: Front Cell Infect Microbiol. 2023 Jan 31;13:1013842. doi: 10.3389/fcimb.2023.1013842 (PMC9927221; doi:10.3389/fcimb.2023.1013842)
Supplement: Supplementary file 1 [file DataSheet_1.docx]

Supplementary Material

**Supplementary table 1. Cytokine concentrations in SK-N-SH conditioned medium, SHRBV virus stock and final concentrations in the diluted virus stock used for macrophage polarization (actual concentration).** Cytokines were quantified in triplicate with the 13-plex Legendplex human antiviral kit, values indicate pg/mL.

|  | IL-1β | IL-6 | TNF-α | IP-10 | IFN-λ1 | IL-8 | IL-12p70 | IFN-α2 | IFN-λ2/3 | GM-CSF | IFN-β | IL-10 | IFN-γ |
| --- | --- | --- | --- | --- | --- | --- | --- | --- | --- | --- | --- | --- | --- |
| Conditioned medium | 4.14 | <0.59 | 0.83 | <0.81 | 4.56 | 976.24 | <0.39 | 0.51 | 13.72 | 0.79 | <8.25 | <0.42 | 1.52 |
| SHRBV stock | 11.65 | 0.74 | 1.62 | 1.6 | 14.72 | 3849.8 | <0.39 | 1.15 | 43.57 | 3.25 | <8.25 | 0.53 | 5.21 |
| Actual concentration | 2.91 | 0.18 | 0.4 | 0.4 | 3.68 | 962.44 | <0.39 | 0.29 | 10.89 | 0.81 | <8.25 | 0.13 | 1.3 |

**Supplementary figure 1. Gene expression validation of a selected set of genes.** Values indicate ΔCt values.


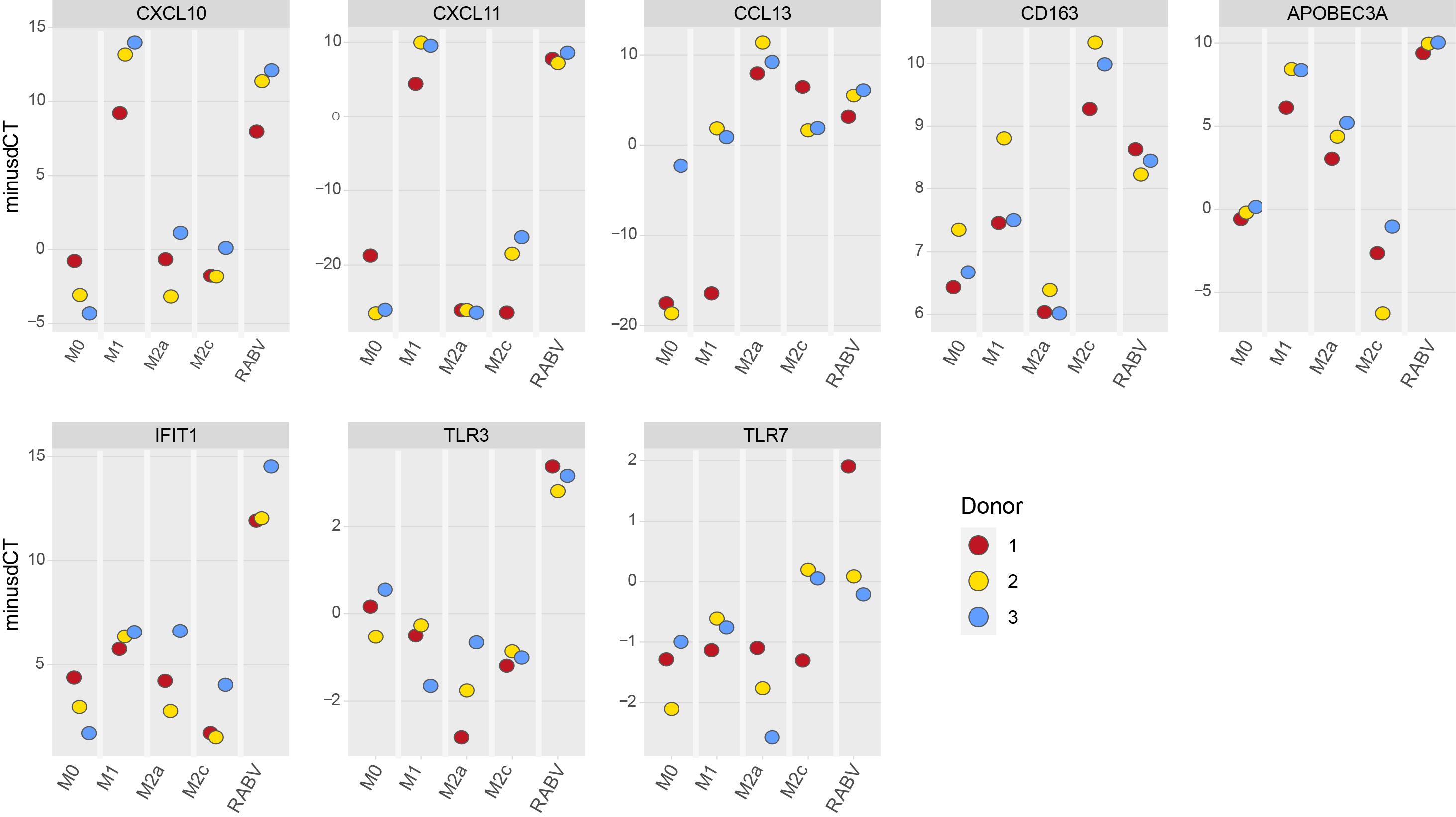


**Supplementary table 2. Primers used for gene expression validation by quantitative PCR**

| **Gene** | **Forward (5' > 3')** | **Reverse (5' > 3')** |
| --- | --- | --- |
| CXCL10 | GTGGCATTCAAGGAGTACCTC | TGATGGCCTTCGATTCTGGATT |
| CXCL11 | GACGCTGTCTTTGCATAGGC | GGATTTAGGCATCGTTGTCCTTT |
| CCL13 | CTCAACGTCCCATCTACTTGC | TCTTCAGGGTGTGAGCTTTCC |
| CD163 | TTTGTCAACTTGAGTCCCTTCAC | TCCCGCTACACTTGTTTTCAC |
| IFIT1 | TTGATGACGATGAAATGCCTGA | CAGGTCACCAGACTCCTCAC |
| APOBEC3A | TGGCATTGGAAGGCATAAGAC | TTAGCCTGGTTGTGTAGAAAGC |
| TLR3 | TTGCCTTGTATCTACTTTTGGGG | TCAACACTGTTATGTTTGTGGGT |
| TLR7 | TCCTTGGGGCTAGATGGTTTC | TCCACGATCACATGGTTCTTTG |
| TBP | GGGGAGCTGTGATGTGAAGT | CCAGGAAATAATTCTGGCTCA |

**Supplementary table 3. Overview of genes uniquely and substantially expressed in RABV macrophages (n=246)**

| AC004024.1 |
| --- |
| AC005514.1 |
| AC006272.1 |
| AC006942.1 |
| AC007684.2 |
| AC007919.2 |
| AC007969.1 |
| AC015802.2 |
| AC018644.1 |
| AC018926.3 |
| AC019322.2 |
| AC022616.4 |
| AC026470.1 |
| AC055822.1 |
| AC068389.3 |
| AC074338.1 |
| AC083900.1 |
| AC089999.2 |
| AC090617.5 |
| AC092634.5 |
| AC093423.1 |
| AC097639.2 |
| AC097641.2 |
| AC098869.1 |
| AC103706.1 |
| AC110792.3 |
| AC112484.4 |
| AC116337.1 |
| AC126120.1 |
| AC129507.1 |
| AC131097.3 |
| AC135721.1 |
| AC234781.4 |
| AC241377.3 |
| AC245884.10 |
| ACIN1 |
| ADD1 |
| ADPRM |
| AL031985.3 |
| AL049873.1 |
| AL118506.1 |
| AL136380.1 |
| AL158801.4 |
| AL162390.1 |
| AL355353.1 |
| AL356273.3 |
| AL357874.2 |
| AL445433.1 |
| AL591516.1 |
| AL596276.2 |
| AL604028.2 |
| AL671277.2 |
| ANG |
| ANO7L1 |
| ANXA2R |
| ARHGAP27 |
| ARMC5 |
| ASB16-AS1 |
| ATF1 |
| ATP5PDP4 |
| AXL |
| BBS4 |
| BLNK |
| BUD13 |
| BX088702.1 |
| C1GALT1 |
| C2CD5 |
| CCDC112 |
| CCP110 |
| CD69 |
| CDKN1C |
| CHROMR |
| CHST12 |
| CHST14 |
| CLECL1 |
| CWC22 |
| DEFB1 |
| DOCK8-AS1 |
| DYNLL1P7 |
| DYRK4 |
| ELOVL3 |
| FAM76B |
| FMC1 |
| FSTL3 |
| FXYD6 |
| GAPT |
| GLRXP1 |
| GMPR |
| GPR141BP |
| GPR155 |
| H2BC11 |
| H2BC5 |
| H4-16 |
| HELQ |
| HELZ2 |
| HESX1 |
| HEXD |
| HMGB1P14 |
| HMGB1P19 |
| HMGN1P28 |
| HMGN2P6 |
| HPSE |
| HSH2D |
| HSPA7 |
| ICAM2 |
| IFITM9P |
| IFNB1 |
| IKBKB |
| IL6ST |
| JPH4 |
| JUP |
| KDM5D |
| KIAA0319L |
| KIN |
| KLK10 |
| KPTN |
| KRI1 |
| KRT8P45 |
| LIN7B |
| LINC00526 |
| LINC02361 |
| LINC02555 |
| LINC02705 |
| LINC02724 |
| LPCAT4 |
| LRRC40 |
| MAGEF1 |
| MCRIP2P1 |
| MED25 |
| MICU1 |
| MIR1289-1 |
| MROCKI |
| MRPS31 |
| MRPS31P1 |
| MTHFSD |
| NAT8 |
| NCOA7 |
| NDUFB8P2 |
| NEK8 |
| NEU3 |
| NEXN |
| NLRX1 |
| NR1D1 |
| ORAI3 |
| ORC4 |
| PARD6A |
| PARP10 |
| PAX8-AS1 |
| PCNPP5 |
| PDCL3P4 |
| PDE1B |
| PHACTR4 |
| PIAS4 |
| PIDD1 |
| PLA2G2D |
| PLAAT3 |
| PLK2 |
| PNRC2P1 |
| PPP1R26 |
| PPP1R2P1 |
| PPP1R3D |
| PRMT9 |
| PRUNE1 |
| PSMC1P5 |
| PTMAP2 |
| PUS7L |
| PXK |
| RAB39A |
| RARRES2P1 |
| RB1 |
| RBM45 |
| RENBP |
| RGS18 |
| RN7SKP150 |
| RN7SKP50 |
| RN7SL253P |
| RN7SL288P |
| RN7SL577P |
| RN7SL615P |
| RN7SL827P |
| RNF8 |
| RNU2-63P |
| RNU2-70P |
| RNU2-72P |
| RNU2-7P |
| RPL30P2 |
| RPL34P33 |
| RPL36AP16 |
| RPS19P7 |
| RPS27P15 |
| RSRP1 |
| SAFB2 |
| SCAMP1-AS1 |
| SCARNA21 |
| SELENOO |
| SELL |
| SEPSECS |
| SESN1 |
| SIGLEC11 |
| SIRT3 |
| SIRT5 |
| SLC15A4 |
| SLC25A30 |
| SNORA71B |
| SNORD91A |
| SNRPCP16 |
| SP3 |
| SPATS2L |
| SPTLC2 |
| ST3GAL5-AS1 |
| STAP1 |
| TENT5A |
| THOC2 |
| TLR3 |
| TLR7 |
| TMEM14DP |
| TNFSF8 |
| TOP3A |
| TPSAB1 |
| TRIM25 |
| TRIM52 |
| TRPM2 |
| U73169.1 |
| UBA52P5 |
| UNC93B1 |
| VAMP1 |
| WAC-AS1 |
| WDSUB1 |
| XPO6 |
| ZBP1 |
| ZIK1 |
| ZKSCAN3 |
| ZNF212 |
| ZNF217 |
| ZNF331 |
| ZNF441 |
| ZNF45 |
| ZNF480 |
| ZNF613 |
| ZNF615 |
| ZNF684 |
| ZNF689 |
| ZNF786 |
| ZNF79 |
| ZNF823 |
| ZNF845 |

**Supplementary table 4. Complete overview of significantly different, or differentially expressed up- and downregulated genes**

| Group | Compared to | Significant different | DEG total | DEG up | DEG down |
| --- | --- | --- | --- | --- | --- |
| M0 | M1 | 5752 | 3934 | 2403 | 1531 |
|  | M2a | 1134 | 851 | 464 | 387 |
|  | M2c | 1048 | 638 | 278 | 380 |
|  | RABV | 4578 | 2773 | 1726 | 1047 |
| M1 | M0 | 5752 | 3934 | 1531 | 2403 |
|  | M2a | 5593 | 3900 | 1783 | 2117 |
|  | M2c | 4892 | 3412 | 1503 | 1909 |
|  | RABV | 2542 | 1654 | 815 | 839 |
| M2a | M0 | 1134 | 851 | 387 | 464 |
|  | M1 | 5593 | 3900 | 2117 | 1783 |
|  | M2c | 1971 | 1352 | 615 | 737 |
|  | RABV | 4421 | 2791 | 1512 | 1279 |
| M2c | M0 | 1048 | 638 | 360 | 278 |
|  | M1 | 4892 | 3412 | 1909 | 1503 |
|  | M2a | 1971 | 1352 | 737 | 615 |
|  | RABV | 3793 | 2366 | 1334 | 1032 |
| RABV | M0 | 4578 | 2773 | 1047 | 1726 |
|  | M1 | 2542 | 1654 | 839 | 815 |
|  | M2a | 4421 | 2791 | 1279 | 1512 |
|  | M2c | 3973 | 2366 | 1032 | 1334 |

**Supplementary table 5. DEGs in M1 when compared to RABV macrophages (A) and DEGs in RABV macrophages when compared to M1 (B).**

A. M1>RABV (n= 363 genes)

| AC004540.2 |
| --- |
| AC004847.1 |
| AC007991.2 |
| AC007991.4 |
| AC016831.4 |
| AC026369.3 |
| AC058791.1 |
| AC245128.3 |
| AC245884.12 |
| ACHE |
| ACOD1 |
| ACP3 |
| ACSL1 |
| ADAM28 |
| ADGRE1 |
| AIFM2 |
| AK3 |
| AKR1B1 |
| AL157871.3 |
| ALAS1 |
| ALDH2 |
| AMPD3 |
| ANKRD22 |
| ANTXR2 |
| APOL1 |
| APOL2 |
| APOL3 |
| APOL4 |
| APOL6 |
| APOO |
| ARID5B |
| B4GALT1 |
| B4GALT5 |
| BATF3 |
| BCL2L14 |
| BTG1 |
| C12orf57 |
| C15orf48 |
| C19orf12 |
| C1orf122 |
| C1QB |
| C1R |
| C1RL |
| C1S |
| C2 |
| C5orf15 |
| CA11 |
| CALHM6 |
| CASP7 |
| CBLN3 |
| CBX6 |
| CCDC115 |
| CCDC50 |
| CCL15 |
| CCL19 |
| CCL23 |
| CCL5 |
| CCNA1 |
| CCR7 |
| CD274 |
| CD33 |
| CD40 |
| CD44 |
| CDC42EP2 |
| CDKN1A |
| CDKN2D |
| CEACAM21 |
| CEACAM3 |
| CEACAM4 |
| CELF1 |
| CES1P1 |
| CFB |
| CFLAR |
| CHI3L2 |
| CHST2 |
| CISH |
| CLCF1 |
| CLDN7 |
| CLEC4D |
| CLEC4E |
| CLEC6A |
| CLU |
| CLUHP3 |
| CP |
| CPD |
| CRISPLD2 |
| CSF2RB |
| CSF3 |
| CTDSP2 |
| CTLA4 |
| CTSO |
| CUL1 |
| CXCL1 |
| CXCL12 |
| CXCL2 |
| CXCL3 |
| CXCL5 |
| CXCL8 |
| CXCL9 |
| CYP27B1 |
| CYRIA |
| DAPP1 |
| DENND5A |
| DHTKD1 |
| DRAM1 |
| DTX2 |
| EBI3 |
| EDN1 |
| EHD1 |
| EPM2AIP1 |
| EPOP |
| ERAP2 |
| EREG |
| ERLIN1 |
| ETV7 |
| FAM177A1 |
| FCAR |
| FCGR1A |
| FCGR1B |
| FCGR1CP |
| FLOT2 |
| FPR2 |
| FTH1P20 |
| FZD1 |
| G0S2 |
| GADD45B |
| GADD45G |
| GBP1 |
| GBP1P1 |
| GBP2 |
| GBP3 |
| GBP4 |
| GBP5 |
| GCC2 |
| GCH1 |
| GGT5 |
| GIMAP7 |
| GIMAP8 |
| GJB2 |
| GK |
| GK4P |
| GNG11 |
| GNS |
| GPR132 |
| GPR157 |
| GPR84 |
| GRAMD1A |
| GYPC |
| GZMB |
| H6PD |
| HAPLN3 |
| HIF1A |
| HLA-DOA |
| HLA-DOB |
| HLA-DPB1 |
| HLA-DQA1 |
| HLA-DQA2 |
| HLA-DQB1 |
| HS3ST3B1 |
| HSD11B1 |
| HSPA2 |
| ICAM1 |
| IDO1 |
| IER3 |
| IGFLR1 |
| IGHEP2 |
| IL10RA |
| IL13RA1 |
| IL15 |
| IL15RA |
| IL1B |
| IL27 |
| IL2RA |
| IL32 |
| IL3RA |
| INHBA |
| INSIG2 |
| IRAK3 |
| IRF1 |
| IRF3 |
| IRF4 |
| ITGB2-AS1 |
| ITGB8 |
| ITPRIPL2 |
| JAK3 |
| KCNE5 |
| KCNJ2 |
| KIAA0040 |
| KLHDC7B-DT |
| KYNU |
| LAMB3 |
| LAMP3 |
| LGALS17A |
| LILRA1 |
| LILRA4 |
| LILRP2 |
| LIMK2 |
| LMNB1 |
| LRP12 |
| LRRC61 |
| LRRK2 |
| LSS |
| LYPD3 |
| LYZ |
| MAP1LC3A |
| MAP3K7CL |
| MAP3K8 |
| MARCHF9 |
| MARCKSL1 |
| MCL1 |
| MCMBP |
| MCOLN2 |
| MLLT11 |
| MMP14 |
| MSANTD3 |
| MSC |
| MT-TV |
| MT1A |
| MT1B |
| MT1DP |
| MT1E |
| MT1F |
| MT1G |
| MT1H |
| MT1HL1 |
| MT1JP |
| MT1L |
| MT1M |
| MT1P3 |
| MT1X |
| MT2A |
| MT2P1 |
| MTF1 |
| MTHFD2 |
| MTMR11 |
| MYO1G |
| N4BP2L1 |
| NAMPT |
| NAMPTP1 |
| NBN |
| NCF1 |
| NCF1B |
| NCF1C |
| NDRG1 |
| NEMP1 |
| NFKB2 |
| NFKBIA |
| NFKBIZ |
| NIPAL3 |
| NKG7 |
| NLRC5 |
| NUAK2 |
| NUB1 |
| OR2I1P |
| OSM |
| P2RX7 |
| PAG1 |
| PDP1 |
| PDPN |
| PFKFB3 |
| PIM1 |
| PLA1A |
| PLAAT4 |
| PLAUR |
| PNRC1 |
| POLB |
| PPA1 |
| PSMB10 |
| PSME2 |
| PSME2P1 |
| PSME2P2 |
| PSTPIP2 |
| PTGER4 |
| PTGES |
| PTGIR |
| PTGS2 |
| PTPN2 |
| PVR |
| QSOX1 |
| RAB29 |
| RAP2C |
| RCAN1 |
| RCN1 |
| RCN1P2 |
| RHBDF2 |
| RHOH |
| RHOU |
| RILPL2 |
| RIPK2 |
| RNF19B |
| RNF24 |
| S100A12 |
| S100A8 |
| SAA1 |
| SCARF1 |
| SCN1B |
| SDC4 |
| SELENOM |
| SEMA4D |
| SERPINA1 |
| SERPINB2 |
| SERPINB9 |
| SERPING1 |
| SLAMF1 |
| SLAMF7 |
| SLC2A6 |
| SLC30A1 |
| SLC31A2 |
| SLC35C2 |
| SLC39A8 |
| SLC6A12 |
| SLC7A11 |
| SMAP2 |
| SMCO4 |
| SNHG15 |
| SNX10 |
| SOCS1 |
| SOCS3 |
| SOD2 |
| SPINT2 |
| SSTR2 |
| ST6GALNAC2 |
| STAP2 |
| STAT4 |
| STBD1 |
| STING1 |
| STK26 |
| STOM |
| STX11 |
| SUSD6 |
| TAGAP |
| TAP1 |
| TAP2 |
| TAPBP |
| TIFA |
| TLR8 |
| TMEM176B |
| TMEM38B |
| TMEM41A |
| TMEM63B |
| TNF |
| TNFAIP2 |
| TNFAIP6 |
| TNIP1 |
| TP53INP2 |
| TRAF1 |
| TREM1 |
| TRGV5P |
| TSC22D1 |
| TSFM |
| TUT1 |
| TXN |
| TXNP4 |
| TXNP5 |
| UBD |
| VAMP5 |
| VILL |
| WARS1 |
| WFDC21P |
| WNT5A |
| ZBED6CL |
| ZC3H12A |
| ZFP36 |

B. RABV>M1 (n=134 genes)

| ABI3 |
| --- |
| ACE |
| AL031985.3 |
| AL158206.1 |
| ANG |
| ANO7L1 |
| APOBEC3A |
| ARL4C |
| AXL |
| B3GNT7 |
| BLZF1 |
| BST2 |
| CBR1 |
| CCDC112 |
| CCL13 |
| CCP110 |
| CD163 |
| CD69 |
| CHST12 |
| CLEC7A |
| CMPK2 |
| CTSC |
| CTSL |
| CXCR2P1 |
| CXCR4 |
| CXorf21 |
| DDX58 |
| DEFB1 |
| DPP4 |
| EIF2AK2 |
| FCGR2C |
| FCGR3A |
| FCGR3B |
| FPR3 |
| FSTL3 |
| FXYD6 |
| GAL3ST4 |
| GMPR |
| GNGT2 |
| GPR155 |
| H2AW |
| HELZ2 |
| HERC5 |
| HERC6 |
| HESX1 |
| HPSE |
| HS3ST1 |
| HSH2D |
| HSPA6 |
| ICAM2 |
| ID3 |
| IFI44 |
| IFIT1 |
| IFIT2 |
| IFIT3 |
| IFITM2 |
| IFITM3 |
| IL10 |
| IRF7 |
| ISG15 |
| JUP |
| KLK10 |
| LACC1 |
| LGMN |
| LGMNP1 |
| LILRA5 |
| LINC01506 |
| LPAR6 |
| LY6E |
| MAFB |
| MASTL |
| MGAT4A |
| MNDA |
| MPEG1 |
| MS4A6A |
| MX1 |
| MX2 |
| NAPSB |
| NCOA7 |
| NEXN |
| NR1D1 |
| NT5C3A |
| OAS1 |
| OAS2 |
| OAS3 |
| OASL |
| OLFML2B |
| OLR1 |
| P2RY6 |
| PDE1B |
| PDK4 |
| PHACTR4 |
| PIK3CD-AS1 |
| PLAAT3 |
| PLK2 |
| PMP22 |
| PNPT1 |
| QPRT |
| RABAC1 |
| RIN1 |
| RPL7AP64 |
| RTCB |
| SAMD9 |
| SCAMP1-AS1 |
| SDC3 |
| SELENOO |
| SELL |
| SHFL |
| SIDT2 |
| SIGLEC11 |
| SIGLEC12 |
| SLC38A5 |
| SLFN12 |
| SPATS2L |
| SSB |
| STAP1 |
| SUGCT-AS1 |
| TENT5A |
| TIFAB |
| TLR3 |
| TMEM123 |
| TNFSF13 |
| TOR1B |
| TRIM14 |
| UBA7 |
| UNC93B1 |
| USP18 |
| VMO1 |
| VSIG10L |
| XPO6 |
| ZBP1 |
| ZKSCAN4 |
| ZNF331 |
| ZNF350 |

**Supplementary table 6. Overview of upregulated and substantially expressed DEGs of RABV macrophages (n=602).**

ABI3

AC005083.1

AC007036.1

AC009303.4

AC010654.1

AC022415.1

AC087741.1

AC093583.1

AC116407.2

AC124319.3

AC127502.2

AC243960.3

ACAP1

ACE

ACOD1

ACOT9

ACP2

ACVRL1

ADA

ADAM8

ADAMDEC1

ADAMTSL4

ADAR

ADGRE1

ADM

AF111167.2

AGTRAP

AIM2

AL021707.6

AL031985.1

AL031985.3

AL158206.1

AL357054.4

AL645933.2

ANG

ANKRA2

ANO7L1

ANXA2R

AOAH

AP002358.2

APBA3

APBB3

APOBEC3A

APOBEC3F

APOBEC3G

APOBEC3H

APOL1

APOL3

APOL6

ARHGEF10L

ARID5A

ARL4C

ARMCX2

ARRDC3

ATF5

ATOX1

ATP13A2

AXL

AZI2

B3GNT7

BATF

BATF2

BDH2

BIRC3

BISPR

BLZF1

BST2

BTG1

BTN2A2

BTN3A1

BTN3A3

C11orf71

C15orf48

C1QA

C1QB

C1QC

C1R

C1S

C2

C3

C3AR1

C9orf72

CALHM6

CARD16

CASP1

CASP10

CASP4

CBR1

CCDC112

CCDC28B

CCL13

CCL18

CCL2

CCL20

CCL23

CCL4

CCL4L2

CCL5

CCL7

CCL8

CCP110

CCR7

CD14

CD163

CD1D

CD209

CD274

CD300E

CD38

CD40

CD47

CD69

CD72

CD80

CD81

CD86

CDC42EP1

CDC42EP2

CDKN1C

CEBPD

CES1

CFB

CGAS

CH25H

CHMP5

CHRNB1

CHST12

CHST14

CIITA

CKB

CLDN23

CLEC10A

CLEC4E

CLEC7A

CMKLR1

CMPK2

CMTR1

CNP

CRAT

CREG1

CREM

CSF2RA

CSF2RB

CSRNP1

CTSC

CTSL

CXCL1

CXCL10

CXCL11

CXCL12

CXCL2

CXCL3

CXCL5

CXCL8

CXCR2P1

CXCR4

CXorf21

CYRIA

CYTIP

DAPP1

DCP1A

DDX58

DDX60

DDX60L

DEFB1

DHX58

DOCK8-AS1

DPP4

DSTNP2

DTX3L

DUSP1

DYNLT1

EBI3

EIF2AK2

ELOVL3

ENPP4

EPSTI1

ETV7

FAS

FBXO6

FCGR1B

FCGR2C

FCGR3A

FCGR3B

FDXR

FFAR2

FGD2

FGD3

FGL2

FKBP5

FKBPL

FMC1

FPR1

FPR2

FPR3

FSTL3

FUT4

FUT7

FXYD6

G0S2

GADD45B

GAL3ST4

GBP1

GBP1P1

GBP2

GBP3

GBP4

GBP5

GIMAP1

GIMAP2

GIMAP4

GIMAP6

GIMAP7

GIMAP8

GJB2

GLRX

GLUL

GMPR

GNA15

GNGT2

GP1BA

GPBAR1

GPR132

GPR155

GPR35

GPR84

GRINA

GSDMD

GTPBP1

H1-0

H2AC18

H2AC19

H2AC6

H2AW

H2BC11

H2BC21

H2BC4

H2BC5

H4-16

H4C8

HAPLN3

HBP1

HCP5

HELZ2

HERC5

HERC6

HESX1

HEXD

HLA-DQA1

HLA-DQA2

HLA-E

HLA-F

HLA-J

HPSE

HS3ST1

HSH2D

HSPA1B

HSPA6

ICAM1

ICAM2

ID3

IDO1

IFI16

IFI27

IFI30

IFI35

IFI44

IFI44L

IFI6

IFIH1

IFIT1

IFIT2

IFIT3

IFIT5

IFITM1

IFITM2

IFITM3

IFITM3P2

IFITM3P3

IFNGR2

IGFBP4

IGFLR1

IL10

IL10RA

IL12RB1

IL15RA

IL18

IL1B

IL27

IL32

IL4I1

IL6

IL6ST

IL7R

IRF1

IRF2

IRF7

IRF9

ISG15

ISG20

ITM2B

JAK3

JUP

KIAA0040

KLHDC7B

KLHDC7B-DT

KLHDC8B

KLK10

KYNU

LACC1

LAP3

LCP2

LGALS2

LGALS3BP

LGMN

LGMNP1

LILRA5

LILRB1

LILRB2

LILRB4

LIMK2

LINC00324

LINC01094

LINC01506

LINC01914

LINC02528

LINC02555

LMBR1L

LPAR6

LRG1

LRP10

LY6E

LYN

LYSMD2

MAFB

MAGEF1

MAP1LC3A

MAP3K8

MARCKS

MARCKSL1

MASTL

MCL1

MCOLN2

MDK

MEPCE

MFSD13A

MGAT4A

MGC16275

MIIP

MIR3945HG

MLLT6

MMP14

MNDA

MOV10

MPEG1

MROCKI

MS4A6A

MS4A7

MSL3

MT1G

MT1H

MT1M

MT1X

MT2A

MX1

MX2

MXD1

MYO1G

N4BP2L1

NADK

NAMPT

NAMPTP1

NAPA

NAPSB

NAT8

NAT8B

NBN

NCF1

NCF1B

NCF1C

NCOA4

NCOA7

NECTIN2

NEK8

NEURL3

NEXN

NFAM1

NFKB2

NFKBIA

NINJ1

NKG7

NMI

NOD2

NR1D1

NRSN2

NT5C3A

NT5C3AP1

NUB1

NUPR1

OAS1

OAS2

OAS3

OASL

OCEL1

ODF3B

OLFML2B

OLR1

OPTN

OSM

OTUD1

P2RY6

PANX1

PARP10

PARP14

PARP6

PARP9

PCGF5

PDCD1LG2

PDE1B

PDK4

PELI1

PFKFB3

PGGHG

PHACTR4

PHF11

PIK3CD-AS1

PILRA

PIM3

PLA2G2D

PLA2G4C

PLAAT3

PLAAT4

PLK2

PLSCR1

PML

PMP22

PNPT1

PPA1

PPM1K

PSMB9

PSME1

PSME2

PSME2P1

PSME2P2

PSTPIP2

PTAFR

PTGER2

PTGIR

QPCT

QPRT

RAB24

RAB9A

RABAC1

RBCK1

RBP1

RCAN1

RFX5

RGL1

RGS2

RHEBL1

RIN1

RNF138

RNF144B

RNF213

RPARP-AS1

RPL7AP64

RPS27P16

RSAD2

RTCB

RTL5

RTP4

S100A8

SAMD9

SAMD9L

SAMSN1

SAT1

SCAMP1-AS1

SCIMP

SDC3

SECTM1

SELENOO

SELL

SEMA4A

SERPINA1

SERPINB9

SERPINF1

SERPING1

SHFL

SHISA5

SIDT2

SIGLEC1

SIGLEC10

SIGLEC11

SIGLEC12

SIGLEC14

SIRPB2

SKIL

SLAMF7

SLC25A28

SLC2A6

SLC31A2

SLC38A5

SLC43A2

SLFN12

SLFN5

SMAP2

SMPDL3A

SNX10

SOCS1

SOCS3

SOD2

SP100

SP110

SPATS2L

SPPL2A

SPTLC2

SSB

ST8SIA4

STAC3

STAP1

STAT1

STAT2

STAT4

STING1

STK17B

STOM

STX11

SUGCT-AS1

SYNGR2

SYT11

TAGAP

TAP1

TAP2

TCN2

TENT5A

TFEC

TGFBI

TIFAB

TLNRD1

TLR2

TLR3

TLR7

TLR8

TMEM123

TMEM140

TMEM171

TMEM176A

TMEM176B

TMEM268

TNF

TNFAIP3

TNFAIP6

TNFAIP8

TNFSF10

TNFSF13

TNFSF13B

TNIP1

TOMM20P2

TOR1B

TOR3A

TP53I13

TRAF3IP3

TRAFD1

TRIM14

TRIM21

TRIM22

TRIM38

TRIM6

TRPC4AP

TRPM2

TSC22D1

TSC22D3

TSPAN3

TYMP

UBA7

UBE2L6

UBE2Z

UNC93B1

USP18

USP30-AS1

VAMP5

VCPIP1

VMO1

VSIG10L

VSIR

WAS

XAF1

XPO6

XRN1

ZBED1

ZBP1

ZC3H12A

ZKSCAN4

ZNF230

ZNF331

ZNF350

ZNF684

ZNF689

ZNF766

ZNFX1

ZSCAN16

ZS
